# A protein interaction based model for schizophrenia study

**DOI:** 10.1186/1471-2105-9-S12-S23

**Published:** 2008-12-12

**Authors:** Pei-Chun Hsu, Ueng-Cheng Yang, Kuan-Hui Shih, Chih-Min Liu, Yu-Li Liu, Hai-Gwo Hwu

**Affiliations:** 1 Institute of Biomedical Informatics, National Yang-Ming University, Taiwan; 2 Center for Systems and Synthetic Biology, National Yang-Ming University, Taiwan; 3 Department of Psychiatry, National Taiwan University Hospital and National Taiwan University College of Medicine, Taipei, Taiwan; 4 Division of Mental Health and Substance Abuse Research, National Health Research Institute, Taipei, Taiwan

## Abstract

**Background:**

Schizophrenia is a complex disease with multiple factors contributing to its pathogenesis. In addition to environmental factors, genetic factors may also increase susceptibility. In other words, schizophrenia is a highly heritable disease. Some candidate genes have been deduced on the basis of their known function with others found on the basis of chromosomal location. Individuals with multiple candidate genes may have increased risk. However it is not clear what kind of gene combinations may produce the disease phenotype. Their collective effect remains to be studied.

**Results:**

Most pathways except metabolic pathways are rich in protein-protein interactions (PPIs). Thus, the PPI network contains pathway information, even though the upstream-downstream relation of PPI is yet to be explored. Here we have constructed a PPI sub-network by extracting the nearest neighbour of the 36 reported candidate genes described in the literature. Although these candidate genes were discovered by different approaches, most of the proteins formed a cluster. Two major protein interaction modules were identified on the basis of the pairwise distance among the proteins in this sub-network. The large and small clusters might play roles in synaptic transmission and signal transduction, respectively, based on gene ontology annotation. The protein interactions in the synaptic transmission cluster were used to explain the interaction between the NRG1 and CACNG2 genes, which was found by both linkage and association studies. This working hypothesis is supported by the co-expression analysis based on public microarray gene expression.

**Conclusion:**

On the basis of the protein interaction network, it appears that the NRG1-triggered NMDAR protein internalization and the CACNG2 mediated AMPA receptor recruiting may act together in the glutamatergic signalling process. Since both the NMDA and AMPA receptors are calcium channels, this process may regulate the influx of Ca^2+^. Reducing the cation influx might be one of the disease mechanisms for schizophrenia. This PPI network analysis approach combined with the support from co-expression analysis may provide an efficient way to propose pathogenetic mechanisms for various highly heritable diseases.

## Background

Schizophrenia is a severe mental disorder with grave personal and social costs [1]. Approximately 1% of the population develops schizophrenia during their lifetime. Over the years, many genes have been reported to be responsible for the susceptibility to schizophrenia [2]. In general, schizophrenia is considered to be a complex disease with multiple genetic and environment etiological factors. Linkage analysis, association and positional cloning studies and candidate gene approaches [3] have been successful in identifying risk genes. The way in which multiple genes, each possibly having a small individual contribution, leads to vulnerability and then the pathophysiology, remains to be elucidated. In order to figure out the relationship among those genes, we should investigate not only in gene-gene interaction level but also a whole picture at the protein level. Recent works to map the protein-protein interaction (PPI) in human to curate human metabolism and regulatory networks offer the relationships among different disease genes [4,5]. The protein clusters in the network may represent the modules with biological functions [6]. It is also reported that if the disease candidate genes are treated as a phenotype, these genes are likely to be function together in the normal cell [7].

In this study, we provided a novel strategy by taking advantages of PPI to discover the regulatory mechanisms among disease candidate genes. We speculated that disease candidate genes may cluster together in a functional network at a protein level. Protein complexes interact with preferred partners to form a biological module serving a specific collective function [8]. When using a network-clustering method by calculating the pairwise distance in the protein interaction network [6], two major protein clusters were found which were involved in synaptic transmission and signal transduction protein cluster. We proposed a model to explain the interaction between NRG1 and CACNG2 which not only fell into the synaptic transmission cluster at protein interaction level but also associated at the gene-gene interaction level.

Recent molecular studies implicate neuregulin1 (NRG1) as the most promising risk factor for schizophrenia [9,10]. Liu and colleagues also found suggestive linkage evidence of schizophrenia to loci near NRG1 on chromosome 8p21 in an ethnically distinct Taiwanese sample [11]. There is also evidence that this genetic risk is elevated when accompanied by genetic changes in the gene for ErbB4, one of neuregulin's binding partners. NRG1-mediated ErbB signalling has important roles in neural development [12-14], as well as in the regulation of neurotransmitter receptors thought to be involved in the pathophysiology of schizophrenia [15]. Hahn and colleagues suggest that enhanced endogenous NRG1-ERBB4 signalling may be responsible for N-methyl-D-aspartate receptors (NMDARs) hypofunction of the disease state [16]. NMDA receptors are a major subtype of glutamate receptors and mediate slow excitatory postsynaptic potentials (EPSPs). Glutamate is the major excitatory neurotransmitter in the brain, and it has been proposed that disruption in glutamate signalling may underlie many of the symptoms of schizophrenia [17]. NRG1 reduces the tyrosine phosphorylation of NMDA receptors, a modification that is triggered by the binding of NMDA or glutamate. NMDAR hypofunction may contribute to the symptomatic features of schizophrenia [18]. ERBB4 associates with NMDAR via DLG4 (also called PSD95), and the binding to DLG4 is probably involved in the enhanced activation of ERBB4. This association provides a physical link between ERBB4 and the NMDAR. These findings add to our basic understanding of glutamatergic transmission, which has been implicated in the pathogenesis of schizophrenia.

CACNG2, also known as stargazin, was found to interact directly with AMPA receptor and allow interaction of the receptor with the scaffold proteins of the postsynaptic density, such as DLG4 [19,20]. In a previous linkage study of schizophrenia that included Taiwanese samples, CACNG2 was also reported as a vulnerability gene for neuropsychologically defined subgroups of schizophrenic patients [21-23]. Bats and colleagues found that a mutation in PDZ domain of CACNG2 will increase AMPA receptor diffusion. CACNG2 regulates trafficking of AMPA-type glutamate receptors and stabilizes them at the postsynaptic density when neurotransmitters are received [20].

## Results and discussion

### Products of candidate disease genes form two major clusters in a schizophrenia-related protein interaction sub-network

**Figure 1:**
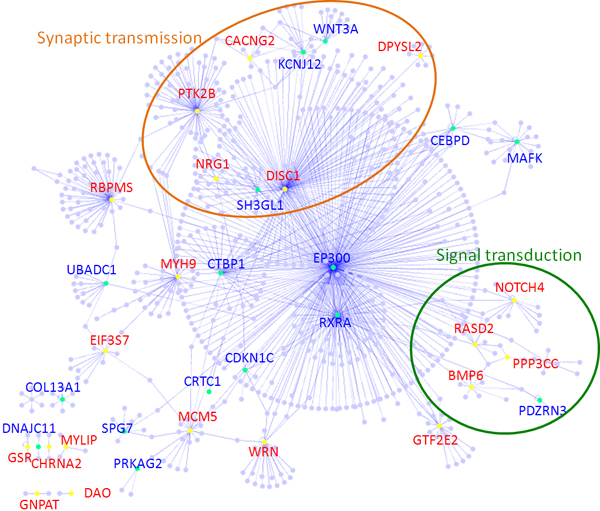
**Protein-protein interaction network and functional cluster annotation**. There were 831 proteins retrieved from IPIR by using 36 candidate genes as the query. The risk genes are closely connected in the protein-protein interaction network. Candidate genes collected from different methods were labeled with different colors. Proteins in big circles mean that they were classified as a cluster by their function.

There are two major types of reactions which are complex formation and covalent modification in the signalling pathway. Both types of reactions have protein-protein interactions (PPI), which can be detected by high throughput methods. It has been shown that proteins which are involved in the same pathway, are likely to cluster together in the PPI network [8]. If the candidate genes may increase the risk of acquiring a disease synergistically, it implies that these genes are likely to work together in the normal cell [7]. Taking these two observations together, it is likely that the products of candidate genes may cluster together in a protein network. Therefore, we have collected 36 reported candidate genes from the literature and used them as a query set to retrieve the nearest neighbours of the candidate proteins. There were in total 831 human proteins retrieved from 6 major PPI databases (see Materials and Methods section). Interestingly, the retrieved interactions linked the gene products of these candidate genes in a big cluster even though these genes were found by a different approach (Figure 1). This result implies that the products of these candidate genes may be important candidates and they will work together in the cell.

**Figure 2:**
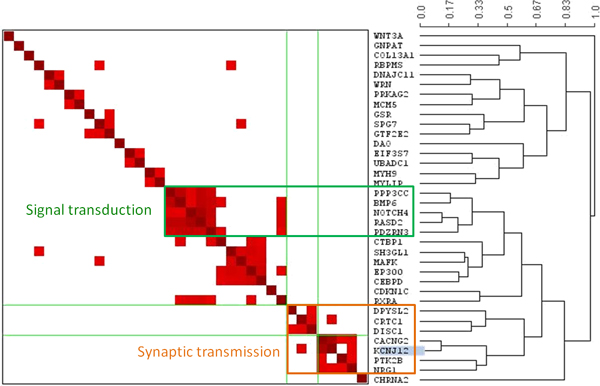
**Protein clustering and classification**. Two major protein clusters were found by using GAP, which were involved with synaptic transmission and signal transduction. The NRG1 and CACNG2 genes were associated not only at the gene-gene interaction level, but also fell into the neuro-transmission cluster on the protein network.

In order to cluster proteins that are close to one another, the distances between every pair of these 831 proteins were computed by using a standard algorithm based on shortest-path of network topology [6]. On the basis of these pairwise distances, two major clusters (Figure 2) were found by using a visualization tool, called Generalized Association Plot (GAP) [24]. By examining the enriched gene ontology terms for the members of these two protein clusters [25,26], the possible function of these clusters was identified. As shown in figure 1, the small cluster (cluster 1), which contains PPP3CC, NOTCH4, RASD2 and BMP6 genes, may be involved in signal transduction. The large cluster (cluster 2), which contains the NRG1 and CACNG2 genes, may be mainly involved in synaptic transmission and sometimes in neural development. NRG1 and CACNG2 were both reported as vulnerability genes from an association study of schizophrenia that included Taiwanese samples [11,22]. These two genes were found to have strong interaction on the basis of linkage and association studies (unpublished data). Thus, it would be interesting to see how these functionally unrelated genes act synergistically in the development of schizophrenia.

**Figure 3:**
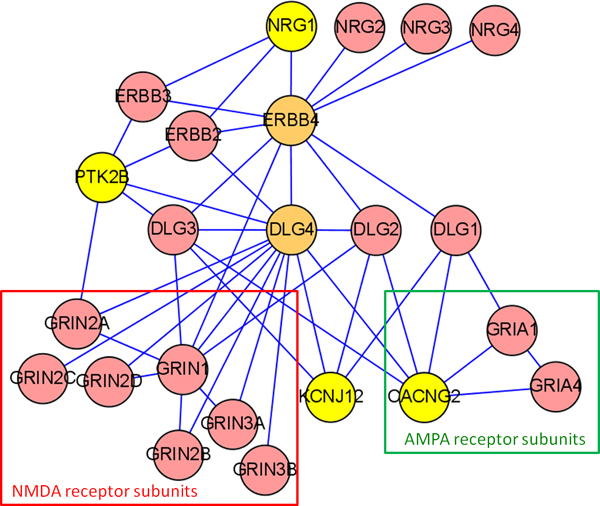
**Sub-network linked NRG1 with CACNG2 according to biological interpretation**. This small network linked NRG1 with CACNG2 via the ERBB4 and DLG4 gene products. DLG4 is interacting with both the ERBB4 and NMDAR gene products. The former is the NRG1 receptor and the latter is a calcium channel triggered by neurotransmitter glutamate. The CACNG2 gene product may recruit another glutamate-triggered calcium channel, AMPA receptor. Proteins painted with yellow color are candidate genes in this study.

Discovering the protein interactions between NRG1 with CACNG2 according to biological interpretationIn order to explore the detailed relation between NRG1 and CACNG2, the cluster 2 sub-network was extracted from the original large network for further study. This cluster contains 7 gene products of candidate genes, which content DPYSL2, CRTC1, DISC1, CACNG2, KCNJ12, PTK2B and NRG1, and 204 interacting proteins; part of this cluster is shown in Figure 3. The NRG1 protein is connected to CACNG2 protein via the ERBB and DLG protein families, which are known to be involved in glutamatergic signalling process [12]. Since cluster 2 proteins were retrieved by the nearest neighbor approach, this subset of proteins will definitely lose some interacting proteins excluded from the sub-network in the disease forming process. Hence, the second or even third neighbors may be needed to propose a biologically plausible mechanism. This step was done manually and the goal is to recover the proteins that may affect synaptic transmission. Therefore, membrane receptors that may link the function of NRG1 and CACNG2 were added to this sub-network. Two major protein families were added, which were the NMDA receptor subunits and the AMPA receptor subunits, respectively. Both receptors are calcium channels that are triggered by glutamate [27]. Thus, they have the potential to act synergistically. DLG4 protein is an important intermediate between NRG1 and CACNG2, because DLG4 protein is interacting with ERBB4, which may receive a signal from NRG1. On the other hand, DLG4 is interacting with both the NMDA and AMPA receptors. As a result, both receptors may receive the NRG1 signal from neighboring neuron cells.

Since functionally related genes are usually co-regulated, we went further to check whether these genes were co-regulated in the brain tissue. If disease is considered as a perturbation to the normal state, different brain tumors may perturb a given gene to different extent. The GSE4271 microarray data set deposited in Gene Expression Omnibus at National Center for Biotechnology Information contains 100 samples from 15 assigned subsets [28]. These gene expression data have been used to compute the correlation of the gene expression for each pair of genes and to establish a relevance network [29]. Since disease is treated as a perturbation, this disease sample-derived network actually represents the co-expression in relation to normal cells.

**Figure 4:**
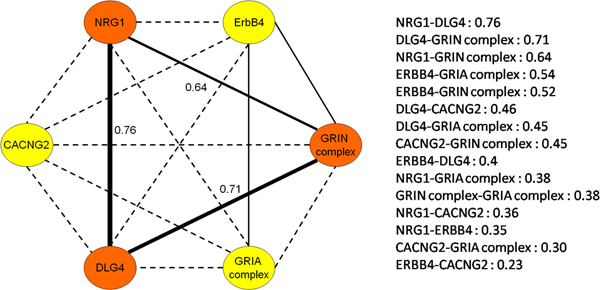
**Impartment component in this network were coregulated at the gene expression level**. Pearson's correlation coefficients between genes were performed. NRG1 and DLG4 showed the strongest correlation 0.76 among these genes. NRG1, DLG4 and NMDA receptor (GRIN complex) genes also showed strong correlation (correlation coefficients above or equal to 0.64) to each other. Black line represent the correlation of gene expression is ≧0.5. The dotted line represent the correlation value is <0.5. These highly correlated gene pairs implies that their protein products may also work together coordinately.

As shown in Figure 4, NRG1, DLG4 and NMDA receptor (GRIN complex) genes showed strong correlation (correlation coefficients above or equal to 0.64). The fact that these gene pairs are correlated implies that their expressed proteins should also be functionally coordinated. The DLG4 gene has a reasonable correlation (coefficient = 0.45) with the CACNG2 and AMPA receptor (GRIA complex) genes. The correlation of gene expression between NRG1 and CACNG2 is less strong (coefficient = 0.36), because CACNG2 may not be the rate-limiting component in the downstream pathway.

### A working hypothesis for interpreting the interaction between NRG1 and CACNG2 genes

As described previously, DLG4 appears to be a hub, which receives the NRG1-ERBB4 signal and then relays the signal to the NMDA receptor and the CACNG2.

**Figure 5:**
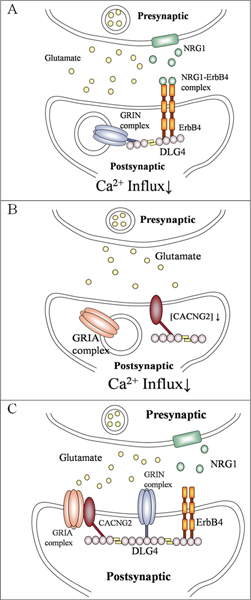
**A glutamatergic synapse focusing on the organization of DLG4 protein**. A) The increased NRG1-ERBB4 signaling may lead to hypophosphorylation of GRIN complex [16] or internalization of GRIN complex [30] in postsynaptic neuron. Less NMDA receptor will be available on the membrane and the capacity for cation influx (mainly calcium influx) will decrease. B) A direct interaction between CACNG2 and DLG4 mediates the synaptic delivery of GRIA complex [38]. The anchored CACNG2 may recruit the GRIA complex to the synaptic region [20] and increase the cation influx. Phosphorylation of DLG4 will release it from the cytoskeleton [32] and fail to recruit the GRIA complex efficiently. C) Taken together, DLG4 links two mechanisms to decrease the cation influx at the synaptic area. The presence of gene variations in both NRG1 and CACNG2 may thus create synergistic effect to affect the influx of Ca^2+^.

Hahn and colleagues suggest that schizophrenia is marked by increased NRG1-ERBB4 signalling and may lead to further suppression of NMDA receptor function by reducing NMDAR tyrosine phosphorylation [16]. Yau and his colleagues demonstrated that the NMDAR in the synaptic sites of ErbB4-deficient mice were more abundant than that of wild-type control and releasing NRG to activate ERBB4 signalling will decrease synaptic activation of NMDA receptors [30,31]. They suggested that this activated ERBB4 signalling will stimulate internalization of NMDA receptors in the synaptic sites. As a result, an increase in the NRG1-ERBB4 signal may either reduce the tyrosine phosphorylation of NMDA receptor or enhance the internalization of NMDA receptor in the postsynaptic neuron (Figure 5A). It is not clear whether these two mechanisms are exclusive at this point. The hypo-phosphorylation event may decrease the glutamate binding and consequently decrease the calcium influx. Alternatively, the internalization of receptor could be mediated by the enhanced interaction between DLG4 and internalized NMDA receptors. Therefore, less NMDA receptor will be available on the membrane and the capacity for cation influx (mainly calcium influx) will decrease.

On the other hand, the interaction between CACNG2 and DLG4 may anchor CACNG2 on the cytoskeleton. The anchored CACNG2 may recruit the AMPA receptor to the synaptic region [20] and increase the cation influx. Phosphorylation of DLG4 will release it from the cytoskeleton [32] and fail to recruit the AMPA receptors efficiently (Figure 5B). Although the calcium-dependent tyrosine kinase PTK2B is interacting with the DLG4 protein (see Figure 3), it is not clear whether PTK2B is catalyzing this reaction. This enzyme has been shown to regulate the activation of calcium channels [33]. Because there are less AMPA receptors in the synaptic area, the capacity for cation influx (mainly calcium influx) will also decrease. Taken together, DLG4 links two mechanisms to decrease the cation influx at the synaptic area. The presence of gene variations in both NRG1 and CACNG2 may thus create a synergistic effect to affect the influx of Ca^2+^(Figure 5C). It has been shown that the schizophrenia patient has more NRG1-ERBB4 complex in the synaptic area than controls. This model specifically predicts that schizophrenia patients will have less cation influx in the synaptic area. Because both NMDA and AMPA receptors are triggered by glutamate, this model also predicts that glutamate may play an important role in pathogenesis. Many of the evidence for the glutamate hypothesis of schizophrenia implicate the NMDA-type glutamate receptor. But the glutamate role may be more complex because there are hints that AMPA receptors also contribute to schizophrenia symptoms, both independently or via effects on NMDA receptors [34].

## Conclusion

DLG4, which receives the NRG1-ERBB4 signal and then relays the signal to the NMDA receptor and the CACNG2, links two mechanisms to decrease the cation influx at the synaptic area. On the basis of the protein interaction network, the NRG1-triggered NMDAR protein internalization and the CACNG2 mediated AMPA receptor recruiting may act together in the glutamatergic signalling process. Since both the NMDA and AMPA receptors are calcium channels, this process may regulate the influx of Ca^2+^. Ca^2+ ^is necessary for transmission at the neuromuscular junction and other synapses. Reducing the synaptic calcium influx due to variants of NRG1 and CACNG2 might explain the basis of schizophrenia. This PPI network analysis approach combined with the support from co-expression analysis may provide an efficient way to propose disease mechanisms for various highly heritable diseases.

## Materials and methods

### Constructing a protein network

Protein-protein interaction data were obtained from Integrated Protein Interaction Resource (IPIR, ). IPIR has integrated protein-protein interaction information from BIND, DIP, HPRD, MINT, MIPS and IntAct databases. In this case, we chose brain, cerebellum, cerebrum and nervous as tissue filter. By using 36 candidate proteins as a data set to look for its primary protein neighbours and secondary protein neighbours, there were 831 proteins retrieved from databases. This network is displayed by Cytoscape which provides basic functionality for a visual representation of the graph and integrated data [35].

### Pairwise distance matrix by generalized association plots (GAP)

For each biological network investigated, relevant proteins (nodes) and the interaction among them (edges) were assembled as follow. Each edge in the network was assigned a length of one. A pairwise distance matrix contains the length of shortest path between every pair of proteins in the network. Each distance in the matrix was shown as an "association", defined as 1/*d*^2^, where *d *is the shortest path distance. Generalized Association Plots (GAP) [24], which is a graphical environment for matrix visualization and information mining, were used to view this results. We have used the Gene Ontology annotations to assign functional category labels to the proteins of the PPI network [25]. GoMiner, a tool for biological interpretation of gene sets, was used to annotate the enriched gene ontology terms of these protein clusters [26]. GoMiner used the Gene Ontology (GO) to identify the biological processes, functions and components represented in gene lists.

### Calculating the correlation values among genes

The microarray data was obtained from Gene Expression Omnibus (GEO) [36], and the accession number for the data series is GSE4271 [28]. Robust Multichip Average (RMA) normalization was performed to compute gene expression values for Affymetrix data and to carry out quality assessment using probe-level metrics [37]. After normalizing the microarray data, we used the Pearson's correlation, performed by a Perl module called "Statistics::RankCorrelation", to represent the correlation coefficient of each pair of probe sets.

## Competing interests

The authors declare that they have no competing interests.

## Authors' contributions

Ueng-Cheng Yang has designed the approaches and made a preliminary analysis. Pei-Chun Hsu has extended the analysis and jointly proposed the working hypothesis with Ueng-Cheng Yang. Kuan-Hui Shih has performed the co-expression analysis for brain tissue by using public microarray data. Hai-Gwo Hwu and his research team have provided the disease candidate genes and the domain knowledge for schizophrenia.
